# Measurement of Fracture Networks in Rock Sample by X-Ray Tomography, Convolutional Filtering and Deep Learning

**DOI:** 10.3390/s25144409

**Published:** 2025-07-15

**Authors:** Alessia Caputo, Maria Teresa Calcagni, Giovanni Salerno, Elisa Mammoliti, Paolo Castellini

**Affiliations:** 1 Department of Industrial Engineering and Mathematical Sciences, Polytechnic University of Marche, 60131 Ancona, Italy; m.t.calcagni@staff.univpm.it (M.T.C.); g.salerno@pm.univpm.it (G.S.); p.castellini@staff.univpm.it (P.C.); 2 Department of Science and Engineering of Matter, Environment and Urban Planning, Polytechnic University of Marche, 60131 Ancona, Italy; e.mammoliti@staff.univpm.it

**Keywords:** 3D X-ray tomography, rock fracture network extraction, U-Net, Gaussian convolution, uncertainty analysis

## Abstract

This study presents a comprehensive methodology for the detection and characterization of fractures in geological samples using X-ray computed tomography (CT). By combining convolution-based image processing techniques with advanced neural network-based segmentation, the proposed approach achieves high precision in identifying complex fracture networks. The method was applied to a marly limestone sample from the Maiolica Formation, part of the Umbria–Marche stratigraphic succession (Northern Apennines, Italy), a geological context where fractures often vary in size and contrast and are frequently filled with minerals such as calcite or clays, making their detection challenging. A critical part of the work involved addressing multiple sources of uncertainty that can impact fracture identification and measurement. These included the inherent spatial resolution limit of the CT system (voxel size of 70.69 μm), low contrast between fractures and the surrounding matrix, artifacts introduced by the tomographic reconstruction process (specifically the Radon transform), and noise from both the imaging system and environmental factors. To mitigate these challenges, we employed a series of preprocessing steps such as Gaussian and median filtering to enhance image quality and reduce noise, scanning from multiple angles to improve data redundancy, and intensity normalization to compensate for shading artifacts. The neural network segmentation demonstrated superior capability in distinguishing fractures filled with various materials from the host rock, overcoming the limitations observed in traditional convolution-based methods. Overall, this integrated workflow significantly improves the reliability and accuracy of fracture quantification in CT data, providing a robust and reproducible framework for the analysis of discontinuities in heterogeneous and complex geological materials.

## 1. Introduction

The structural integrity and functional performance of many natural and engineered materials are strongly influenced by the presence and evolution of internal discontinuities, such as fractures, cracks, and pores [1,2]. These features may originate from natural geological processes, mechanical stress, or material degradation over time and significantly affect mechanical, thermal, and transport properties [3,4]. Their detection and quantitative characterization are therefore essential in numerous domains, including civil and aerospace engineering, geosciences, and materials science [5,6].

Among the many classes of materials where internal defects play a critical role, two stand out for both scientific interest and technological relevance: metallic alloys and natural rocks. In metals, defects such as fatigue cracks or voids can compromise structural durability and service life [7,8]. In geological materials, fracture networks govern key processes such as fluid flow and mechanical behavior in the subsurface [9,10,11].

To analyze such internal structures without damaging the sample, non-destructive evaluation (NDE) techniques are widely used. In metallic materials, high-frequency ultrasonic testing, X-ray computed tomography (CT), and eddy current inspection have proven effective in identifying and monitoring micro-defects. CT, in particular, allows for volumetric imaging with increasing spatial resolution, enabling the detection of small discontinuities even in dense, heterogeneous materials [8,12,13].

In geological materials, understanding the geometry and connectivity of fracture networks is essential for modeling fluid flow, mechanical behavior, and transport properties. Borehole imaging techniques, such as optical and ultrasonic logs, provide local information on fracture orientation and position but are limited to fractures intersecting the borehole [14,15]. While the statistical analysis of borehole data allows for the inference of fracture sets and spacing, reconstructing full 3D networks remains challenging. Deterministic information is confined to sampled regions, and surrounding fractures must be modeled statistically [10,16,17]. Discrete Fracture Network (DFN) models are thus used to stochastically represent these systems based on available statistical descriptors [9].

In this context, 3D X-ray CT provides a powerful, non-destructive technique to visualize internal fracture systems with high spatial resolution [18,19,20], offering key input for DFN modeling and improving the reliability of simulations in reservoir engineering, geothermal energy, and rock mechanics. However, segmenting narrow, planar fractures from CT volumes remains difficult due to the Partial Volume Effect and low contrast between fractures and the rock matrix [21,22]. Standard thresholding techniques often produce inaccurate networks, overestimating connectivity or introducing artificial features [23]. For instance, local thresholding methods such as the Technique of Iterative Local Thresholding introduced by [24] can improve the delineation of individual fractures, but struggle when applied to complex fracture networks. Similarly, Hessian-based filters like Multiscale Hessian Fracture filtering, while effective in enhancing planar structures, often blur fracture edges due to the Gaussian smoothing step, which, in turn, compromises the accurate quantification of fracture surface roughness [25].

In addition, the effectiveness of conventional methods in segmenting intersecting fractures remains limited, particularly when fracture apertures approach or fall below the spatial resolution of the X-ray CT imaging system. In such cases, segmentation performance is highly sensitive to user-defined parameters such as Gaussian filter standard deviation or intensity thresholds, leading to inconsistent results.

To overcome these limitations, recent studies have increasingly focused on machine learning approaches, which offer improved segmentation accuracy and robustness with minimal user intervention. Supervised methods, such as convolutional neural networks and, more specifically, U-Net architectures [26] have demonstrated superior performance in segmenting fine and elongated features from X-ray CT data owing to their hierarchical feature extraction and spatial encoding–decoding capabilities.

In this study, two segmentation strategies were compared for the extraction of fracture networks from CT datasets. The first was a deterministic image processing pipeline aimed at enhancing fracture visibility and minimizing artifacts caused by noise or thresholding errors. The second involved the application of a U-Net deep learning model, trained to recognize fractures in images, to perform automatic fracture identification on CT slices.

Both approaches generated a 3D point cloud that captured the spatial distribution and orientation of fractures. These point clouds were subsequently processed through a clustering procedure to identify individual fracture planes and their intersections, resulting in a Discrete Fracture Network (DFN) suitable for further quantitative analysis.

While the methods presented are generalizable to a variety of materials, this work focused on rock specimens as a representative case study. However, the same processing framework can be extended to other heterogeneous materials, including metallic alloys, thereby supporting broader research efforts in sustainable mobility and advanced material diagnostics, which are key priorities of current national innovation agendas.

## 2. Materials and Methods

### 2.1. Rock Core Extraction and Discontinuities Description

**Figure 1:**
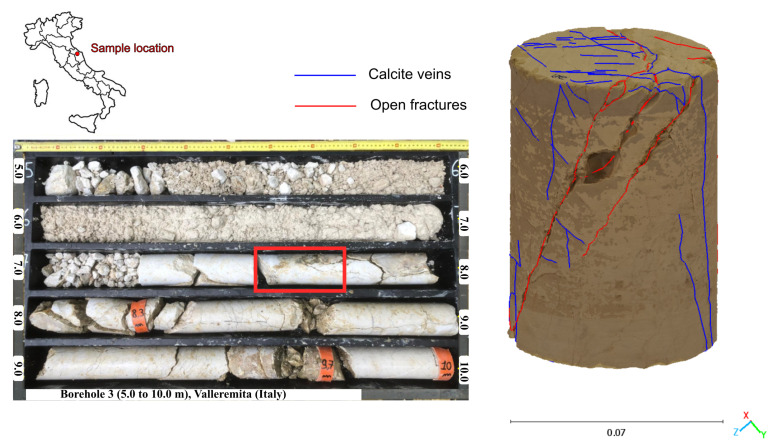
Location and features of the analyzed core fragment. (**Left**) Core box with the selected sample highlighted in red (Valleremita, Marche Region, Italy). (**Right**) A 3D model of the sample showing open fractures (red) and calcite veins (blue).

The analyzed sample was a core fragment from the Jurassic Maiolica Formation (Upper Tithonian–Lower Aptian) within the Umbria–Marche succession of the northern Apennines (Italy), an area shaped by multiple tectonic phases, including extensional, compressional, and recent reactivation events [27,28,29,30,31]. Collected in the Valleremita area (Marche Region, Italy), the sample (Figure 1) was selected for its well-developed open joints, which exhibit an average dip angle of approximately 60°. The rock matrix, composed mainly of calcilutites, also contains calcite-filled veins—with some showing partial dissolution—and minor tectonic stylolites with clay-rich infill. These calcite veins, consisting primarily of fibrous crystals, are interpreted as extensional features formed by fluid migration and subsequent mineral precipitation along pre-existing fractures [32]. The fracture network thus reflects the imprint of multiple tectonic events associated with the Apennine orogeny [27]. Unlike open fractures, identifying calcite veins in carbonate rocks via tomographic methods is challenging due to the similar mineralogical composition of the veins and the host matrix [32,33]. This sample configuration provides an ideal test case for extracting fracture networks using high-resolution imaging techniques.

### 2.2. The 3D X-Ray Tomography

The methodology adopted in this study was based on X-ray CT, a technique that enables the visualization and quantification of an object’s internal density distribution through X-ray radiation [34].

For this analysis, the Zeiss Metrotom 1500 [35] system was employed, which provides a maximum measurement volume of 350 mm in the *x*, *y*, and *z* directions. This system is designed with a fixed tube-to-detector distance of 1500 mm, while allowing for adjustments along the X-axis between the X-ray tube and the center of the rotating platform. Such flexibility enables precise control over voxel size, where decreasing the distance results in finer voxel resolution, reaching an impressive level of detail of up to 5 μm. The ability to fine-tune voxel size is essential as it allows the optimization of imaging parameters to capture intricate details of scanned objects [36].

Tomographic data acquisition consists of two key phases: the scanning process, during which the object is exposed to X-ray beams, and the reconstruction phase, where the acquired data is processed into a three-dimensional model.

During scanning, the X-ray tube emitted a conical beam through the diaphragm, which then passed through the sample before reaching the X-ray detector. Due to the different absorption properties of the materials within the sample, the detector captured a series of grayscale images representing the internal structure. The object was gradually rotated in steps over a full 360-degree cycle, allowing the collection of projections from multiple angles [37].

Following data acquisition, the reconstruction phase was carried out. The numerous two-dimensional projections obtained during scanning were processed and combined to generate a detailed, three-dimensional representation of the rock sample’s internal structure and density distribution.

For this specific analysis, the total acquisition time was approximately 1.5 h, also due to the use of an image averaging setting of 3, which improved the signal-to-noise ratio by reducing random noise in the projections. The scan was performed using the following settings: 185 kV voltage, 591 μA current, 666 ms integration time, and gain 4. These parameters were chosen to ensure sufficient image contrast and minimize noise while maintaining an acceptable acquisition time. The resulting spatial resolution of the reconstructed volume was 70.69 μm, with a final voxel matrix size of 1495×1553×1770 voxels along the *x*, *y*, and *z* directions, respectively, ensuring a highly detailed volumetric representation of the scanned object.

### 2.3. The 3D Fracture Network Extraction

This section presents two distinct approaches for the extraction of 3D fracture networks from cylindrical rock samples imaged through X-ray CT. The entire image processing pipeline and data analysis were implemented using MATLAB. Both methods aimed to identify and isolate fracture geometries within the volumetric data, but they relied on different processing paradigms: one based on deterministic image processing and the other on deep learning.

The first method (deterministic method), developed specifically for this study, utilized a pipeline of traditional image processing steps to enhance and detect fractures. The procedure includesd

- Circular masking of each tomographic slice to isolate the rock matrix;
- Normalization and compensation for radial density gradients;
- Extraction of fracture signatures through 1D density profiles and convolution-based peak detection.

**Figure 2:**
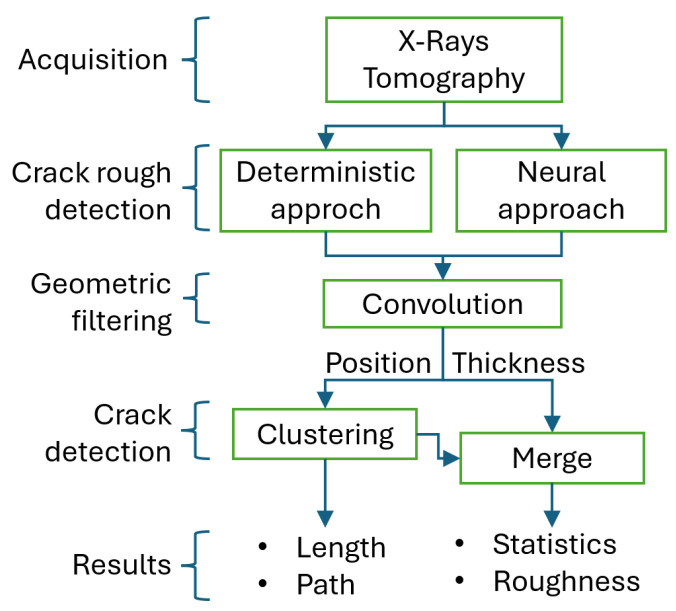
Workflow of the fracture segmentation and 3D analysis.

**Figure 3:**
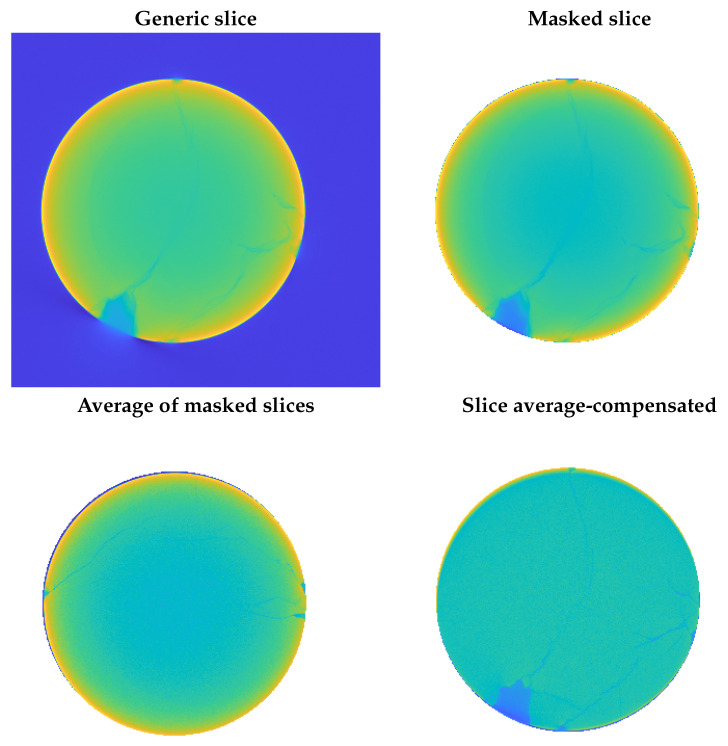
Example of an acquired tomographic slice and related processing steps for crack enhancement.

**Figure 4:**
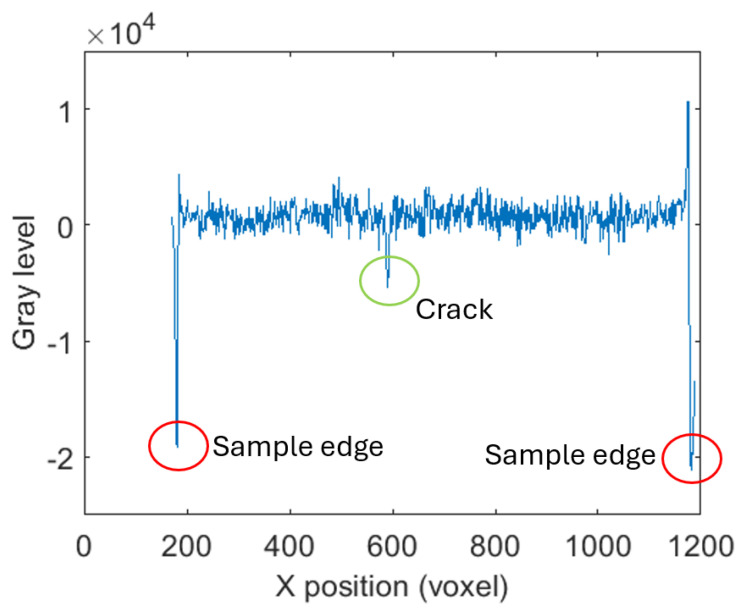
Example of 1D density profile across a tomographic slice, used for peak detection related to fracture locations.

The complete workflow for this method is illustrated in the flowchart in Figure 2, while Figure 3 shows examples of a raw slice, its masked version, and the average-compensated outputs. These steps were designed to isolate density variations linked to fractures while suppressing structural noise or background fluctuations. An example of a 1D density profile used to identify peak-related features is shown in Figure 4. From these 1D density profiles, fractures were detected as local gray-level minima corresponding to air-filled discontinuities within the rock. A dynamic thresholding operation was applied, where pixels with attenuation values below a calibrated threshold—empirically set at approximately 25% below the local normalized mean—were classified as fracture points. The crack width was estimated by counting the number of consecutive sub-threshold pixels and multiplying this by the voxel size. To enhance detection robustness and provide a local aperture measure, this thresholding was embedded within a convolution filter using a square-wave kernel of variable size. This method was applied both row-wise and column-wise to capture fractures in different orientations, with subsequent pruning to eliminate redundant detections. Validation against a subset of fractures manually annotated by an expert yielded a mean absolute error of 0.12 mm in width estimation, demonstrating the approach’s accuracy within the limits of voxel resolution.

The second method (U-Net method) built on a data-driven approach based on the U-Net architecture, a convolutional neural network originally developed for biomedical image segmentation. Here, U-Net was applied to the tomographic slices to generate binary segmentation masks that indicated the presence of fractures.

The complete processing chain is summarized in the flowchart in Figure 2. After segmentation, the binary masks underwent a series of post-processing operations: smoothing, connected component labeling, voxel-to-point cloud conversion, and clustering based on spatial proximity. These steps enabled the reconstruction of the 3D fracture network in terms of discrete voxel clusters, each representing an individual fracture surface or segment.

#### 2.3.1. Method Based on Deterministic Image Processing

**Figure 5:**
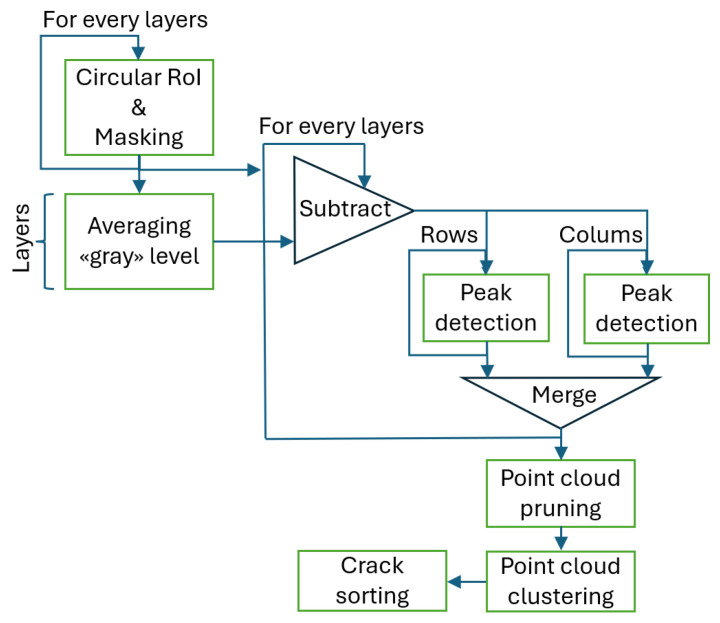
Workflow of the deterministic image processing pipeline for fracture detection.

The specimens analyzed in this study were defined as cylindrical in shape. They were positioned in the tomograph with the main axis of the cylinder oriented in the vertical direction. This configuration represents an anomaly concerning the best practices of tomographic measurement, where installation under symmetrical conditions is typically not recommended. This is due to the risk that the real symmetries present in the object may be confused with the potential symmetries induced by the tomographic algorithm. In this instance, however, the focus of the analysis was directed toward the cracks, which did not exhibit such symmetry. Positioning the cylinder along the vertical axis facilitated the reading and processing of the results. The completed flowchart is shown in Figure 5.

For each individual layer, the circular section of the specimen enabled masking and the accurate alignment of each layer. From these aligned layers, the average image was subsequently derived, representing the density distribution within the specimen, particularly along the radial direction. Analysis of the tomography results revealed variations in density, primarily attributed to three factors:

- Natural density variations occurring within the specimen;
- Apparent density variations associated with the beam hardening effects during scanning;
- Density variations related to the presence of cracks and the air within them.

The first two types of density variations posed a challenge in the recognition of cracks. The first variation manifested locally as certain areas of the specimen could contain inclusions of different materials with varying densities. In contrast, the second variation arose systematically across each individual layer due to beam hardening effects in the scanning process. Because the X-ray source was polychromatic, X-rays passing through thicker regions of the sample were hardened more than those passing through thinner regions. This effect resulted in a cupping artifact, where the center of the specimen appeared less intense. Although there existed standard correction routines to compensate for beam hardening, in this study, the average density distribution was subtracted from each individual layer to mitigate this artifact and enhance the fluctuations associated specifically with cracks.

For each individual layer, the density distribution was examined along the rows and columns, yielding one-dimensional density profiles. Peaks corresponding to minimum density values associated with the presence of air within the cracks were identified along these profiles. Despite the normalization performed on the density profiles, the presence of measurement noise suggested a need for the introduction of a convolutional algorithm for peak detection related to the cracks.

In selecting the convolutional filter, a fundamental decision pertained to its shape. While observation of the density profile at the crack often revealed a Gaussian-like trend, this profile arose not only from the tomograph’s intrinsic resolution limits but also as a consequence of the reconstruction algorithm. Specifically, the filtered back-projection method applied a ramp filter in the frequency domain followed by a Hilbert transform in the spatial domain, which introduced characteristic edge ripples and blurring around sharp discontinuities. Therefore, instead of a Gaussian kernel, a square-wave profile of variable width was selected. Although this profile yielded a lower numerical correlation due to these reconstruction effects, it more accurately represented the true physical geometry of sharp-edged fractures, improving the reliability of fracture detection.

The identified peaks, represented by their XYZ coordinates, could be depicted as point clouds corresponding to the various cracks. This operation was repeated for rows and columns to ensure the identification of cracks regardless of their propagation direction. However, this redundant approach could result in repeated cloud points, as the same crack point could be located from different observation angles.

Consequently, the point cloud obtained necessitated a pruning operation to eliminate redundant points, thereby reducing the overall data volume. Ultimately, the resulting point cloud underwent a clustering operation to identify the most numerous clouds that were representative of cracks, isolate points that were indicative of noise, and separate the various cracks from one another.

#### 2.3.2. Method Based on U-Net Algorithm

U-Net is a fully convolutional network (FCN) that was mainly developed for the segmentation of biomedical images. It was pioneered by Olaf Ronneberger et al. [26]. This structure is primarily used for semantic segmentation, and it consists of a contraction path and an expansion route. The contraction involves the repetitive application of two 3×3 convolutions (a convolution is an integral that conveys the amount of superposition of a function *g*, shifted onto another function *f*), each followed by a rectified linear unit (ReLU) and a 2×2 max pooling operation (used to decrease the spatial size of the input volume) with a two-step down-sampling. At each down-sampling pass, the number of feature channels is duplicated. Each step of the expansion path consists of an up-sampling of the feature map followed by a 2×2 convolution (‘up-convolution’) that halves the number of feature channels. Lastly, a concatenation with the corresponding feature map cropped from the contraction path is performed, followed by two 3×3 convolutions, each followed by a ReLU. Trimming is necessary due to the loss of boundary pixels with each convolution. In the final layer, a 1×1 convolution is used to map each 64-component feature vector to the required number of classes. In total, the network has 23 convolutional layers.

**Figure 6:**
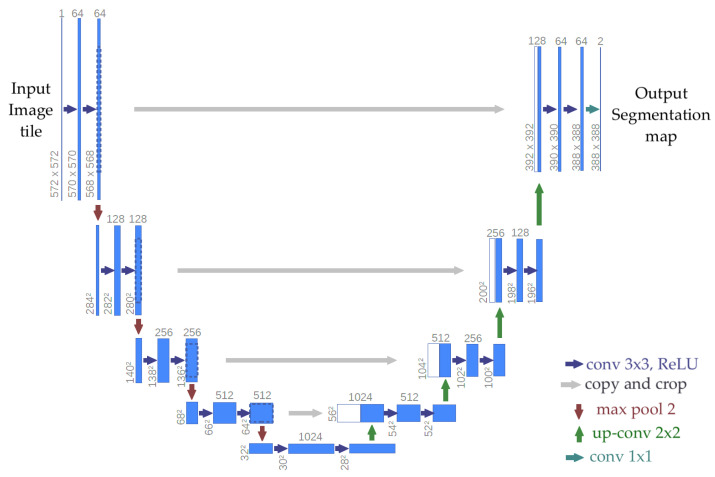
Example of architecture representing the main functionalities of U-Net.

Despite being originally conceived for medical imaging, U-Net is now extensively deployed in various image segmentation challenges due to its effectiveness and outstanding performance with limited training data. Chen et al. proposed an automated detector of bridge failures using the U-Net architecture [8]. An automatic crack detector was developed for masonry structures, confirming the application of this architecture in fields beyond the biomedical sector [38]. An example of the U-Net architecture is shown in Figure 6.

Originally developed for medical image segmentation, U-Net has proven to be effective across various fields. In medicine, U-Net and its variants remain state-of-the-art for image segmentation, with various applications [39,40]. Other applications include the detection of virtual targets on the bodies of professional swimmers for the purpose of trajectory detection and sports performance measurement. In [41], images of breeding crickets were processed to count crickets and identify male from female individuals. In [42], X-ray tomography slices were analyzed to detect the presence of plastic inclusions within fish specimens. The study then led to an analysis of possible causes of measurement and, particularly, processing uncertainty using artificial intelligence techniques presented in [43]. In geology and remote sensing, it has been used to enhance satellite image classification [44,45] for rock skeleton and rock fracture identification [46,47,48].

These works highlight U-Net’s versatility and effectiveness in automated image analysis across disciplines.

The data used for the training, validation and test of the U-Net architecture was a private dataset consisting of 750 images collected from open source online data and data collected privately during the EU project activities carried out over the years. In fact, the Model Loss was considered to consist of the sum of the Dice Loss and Focal Loss. The training took 130 epochs. The dataset was divided into training, validation and testing subsets following the following proportions: 80%, 10%, 10%.

Following the acquisition of segmentation masks generated by the U-Net architecture, a systematic approach was adopted to transition from these masks to the identification of clusters. Initially, the binary segmentation masks underwent a smoothing process to reduce noise and enhance the integrity of the identified features. This pre-processing step was essential for improving the subsequent analysis.

After smoothing, connected component analysis was employed to identify groups of adjacent pixels within the masks that corresponded to fractures in the analyzed volume. The minimum detectable aperture of the crack corresponded to a pixel (dimension of a voxel), but this could not be isolated; otherwise, the algorithm would suppress it. This process facilitated the transformation of the binary masks into a three-dimensional point cloud representation, where each point reflected a voxel associated with a fracture.

To facilitate a detailed examination of the fracture characteristics, the point coordinates were mapped into three-dimensional space. To further refine the analysis, a clustering algorithm based on minimum distance was implemented, specifically utilizing the pcsegdist method (point cloud segmentation by distance), a built-in MATLAB function. This method segments a point cloud into clusters by grouping points that lie within a specified Euclidean distance from each other, effectively identifying spatially distinct features. This clustering phase is crucial for discerning the structural features of fractures, enabling a more comprehensive analysis of their geometry and distribution.

## 3. Results

This section presents the outcomes of two fracture detection methods applied to the tomographic data of the rock core sample: a deterministic image processing technique and U-Net-based deep learning segmentation. Each method produced distinct outputs, which were visually compared to evaluate the effectiveness and completeness of fracture network extraction.

### 3.1. Method Based on Deterministic Image Processing

**Figure 7:**
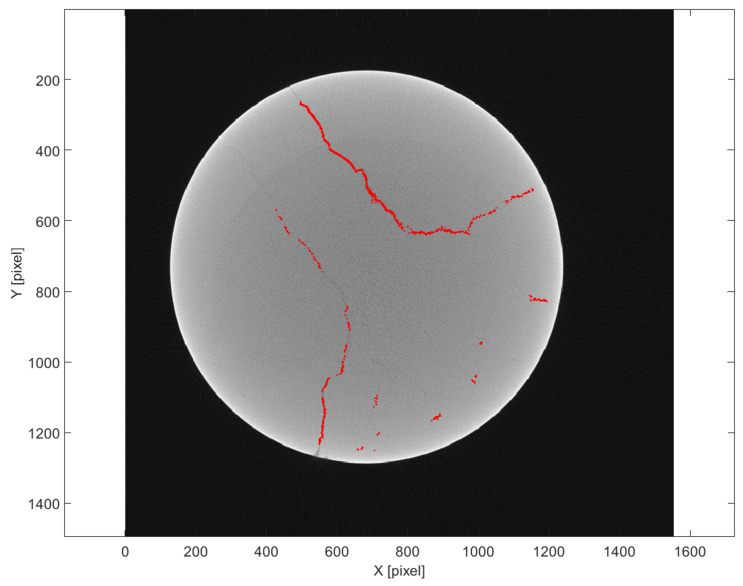
Detected fracture points using deterministic method. Points are projected (in red) onto the corresponding tomographic slice.

Figure 7 displays the results obtained using the deterministic approach based on peak detection in normalized density profiles. The identified fractures are represented as a set of discrete points (in red) superimposed on a tomographic slice of the sample. These points corresponded to local minima in the density profiles, which were assumed to indicate the presence of air-filled fractures. The method successfully identified linear features aligned with visible discontinuities in the rock matrix. However, some noise and redundant points were also evident, especially in regions with lower contrast or overlapping features. The method is effective in capturing high-contrast, open fractures, but may miss or inaccurately localize subtle features or partially filled cracks.

### 3.2. Method Based on U-Net Algorithm

**Figure 8:**
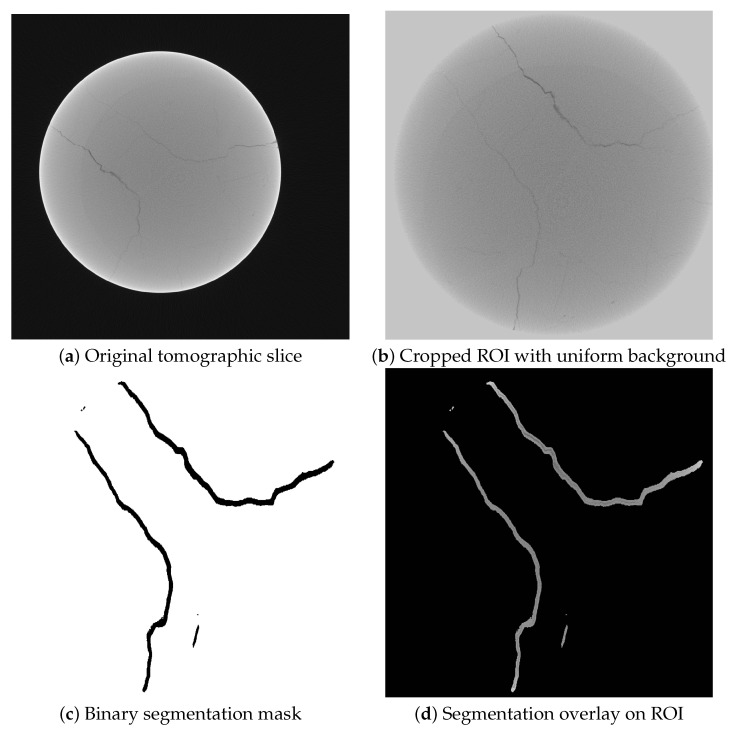
Fracture segmentation workflow using U-Net method. (**a**) shows the original tomographic slice; (**b**) displays the cropped Region of Interest with a uniform background as used for model input; (**c**) shows the binary mask output of the model; (**d**) presents the final overlay of the segmentation mask on the tomographic ROI.

Figure 8 illustrates the segmentation masks generated by the U-Net convolutional neural network: panel (a) shows the original tomographic slice, providing the unprocessed grayscale image of the rock sample; panel (b) displays the cropped Region of Interest (ROI) used as input for the neural network, with the background set to a uniform value to improve model focus on relevant structures; panel (c) presents the binary segmentation mask generated by the U-Net model, where black pixels correspond to regions predicted as fractures and white pixels represent the background; and, finally, panel (d) overlays the binary mask onto the tomographic ROI, highlighting the detected fracture areas directly on the grayscale image, which facilitates the interpretation of fracture morphology. To enhance interpretability, each mask was applied as a binary filter to the corresponding tomographic slice: voxels outside the predicted ROI were set to zero, while the grayscale intensity values were preserved only within the segmented zones. The resulting image shows the internal texture of the tomographic data exclusively where the U-Net identified potential fractures, thereby facilitating a clearer visualization of fracture morphology. Compared to the deterministic method, this approach yielded a more continuous and spatially coherent representation of fractures. The U-Net model successfully detected both prominent and subtle features, demonstrating robustness to variations in density and texture, which was also due to the fact that the model overestimated crack thickness in segmentation.

From the segmentation masks, a post-processing pipeline was applied to extract a three-dimensional point cloud corresponding to fracture voxels. These points were identified using an intensity-based thresholding algorithm, applied exclusively within the ROI defined by the U-Net output. This ensured that the detection process was spatially constrained to regions previously classified as likely fractures by the network.

**Figure 9:**
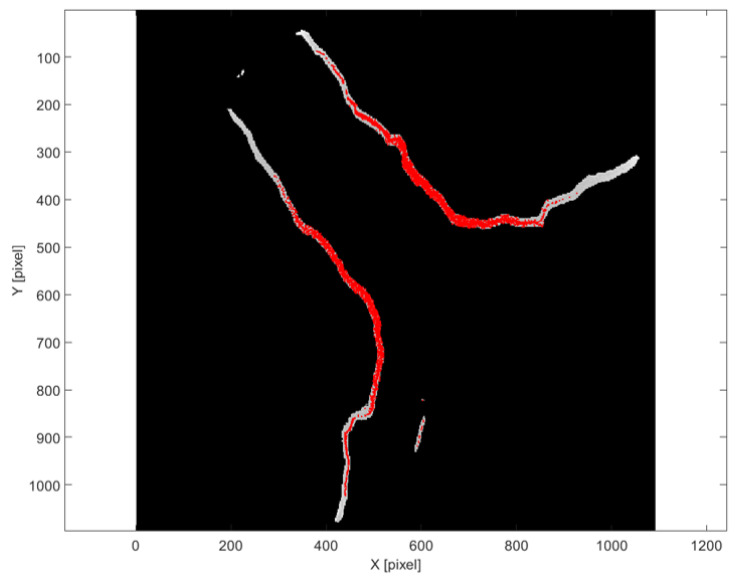
Detected fracture points using U-Net method (red points) are overlaid on the masked clustered tomographic slice, where grayscale values are retained only within the region predicted by the model.

Figure 9 presents the same masked tomographic slice shown in Figure 8, now overlaid with the extracted fracture points (in red). The underlying image retains grayscale information only within the predicted ROI, while all other regions are suppressed. This combined representation allows for a direct comparison between the U-Net segmentation and the outcome of the intensity-based fracture detection, supporting the evaluation of the method’s capability to capture fracture geometry. Despite the spatial constraint imposed by the U-Net masks, the use of intensity thresholding introduced certain limitations. Background noise in the tomographic slices could lead to the inclusion of isolated non-fracture pixels among the detected points. This ambiguity was particularly relevant in regions where the contrast between fractures and the surrounding matrix was low. A trade-off was therefore necessary: lowering the threshold increased the risk of false positives, while raising it could result in the omission of relevant fracture details. The selected threshold was chosen to balance the minimization of false detections with the preservation of the fracture network’s continuity.

### 3.3. Comparative Analysis of Fracture Detection Methods

The two methods for fracture extraction presented distinct characteristics in terms of accuracy, structural coherence, and noise sensitivity. The deterministic method, based on image enhancement and peak detection on normalized density profiles, allowed for the targeted identification of fracture points. Its main strength lay in the precision of point localization, as the algorithm detected density minima strongly associated with air-filled cracks. This resulted in well-defined fracture traces with reduced background noise. However, the method tended to be less effective in capturing the full extent of irregular or low-contrast fractures, leading to sparser point clouds and the potential underestimation of fracture dimensions.

In contrast, the deep learning-based approach generated segmentation masks that more effectively captured the spatial continuity of fractures. The resulting point clouds, derived through thresholding and clustering, were denser and more representative of the global fracture geometry (Figure 9). Although the U-Net output was a binary mask, it may still include noisy or ambiguous voxels that do not clearly represent fractures. Therefore, rather than considering all voxels labeled as fractures, the binary mask was used as an ROI on the original tomographic data. Within this ROI, an intensity-based threshold was applied to isolate voxels that exhibited intensity values typical of fractures. This step helped refine the detection by reducing false positives caused by noise or uncertain segmentation, before proceeding with clustering to identify distinct fracture features. This made the method particularly suited for mapping complex or branching fracture networks. Nevertheless, due to the threshold-based extraction from the segmentation masks, the method was more susceptible to including background noise, especially in low-contrast regions. Some points could correspond to non-fracture voxels, introducing a level of ambiguity. Efforts were made to tune the threshold to minimize false positives without excessively compromising the inclusion of fracture voxels. This trade-off reflects a key limitation of the method when high precision is required at the voxel level.

Overall, the deterministic method favors precision and low noise, whereas the deep learning approach excels in coverage and continuity. A combined use of both methods could potentially leverage their respective advantages, improving the robustness and completeness of fracture network characterization.

A 3D point cloud of the detected fracture voxels was generated from each method. These visualizations provide a spatial overview of the fracture network as identified by the respective approaches.

**Figure 10:**
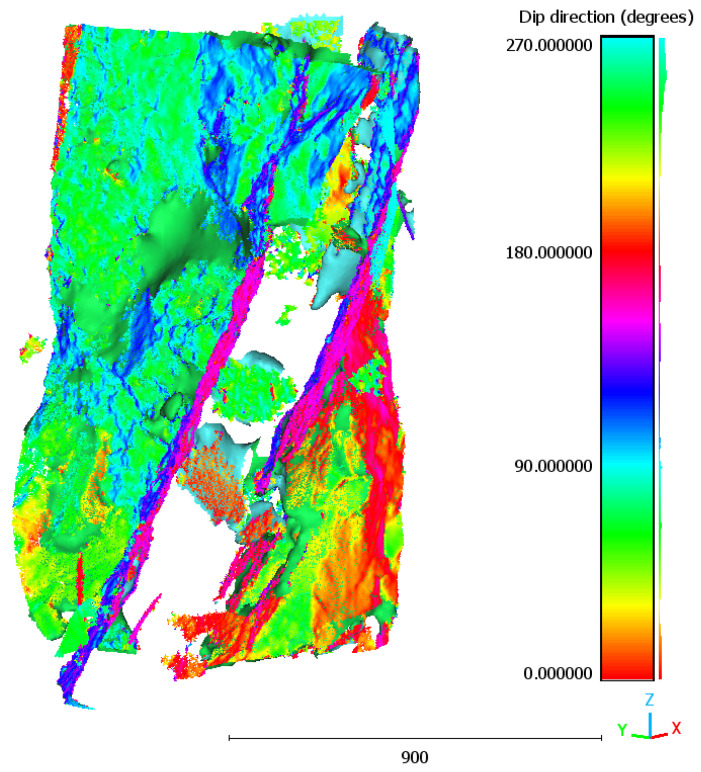
The 3D point cloud of fracture voxels extracted using deterministic method.

**Figure 11:**
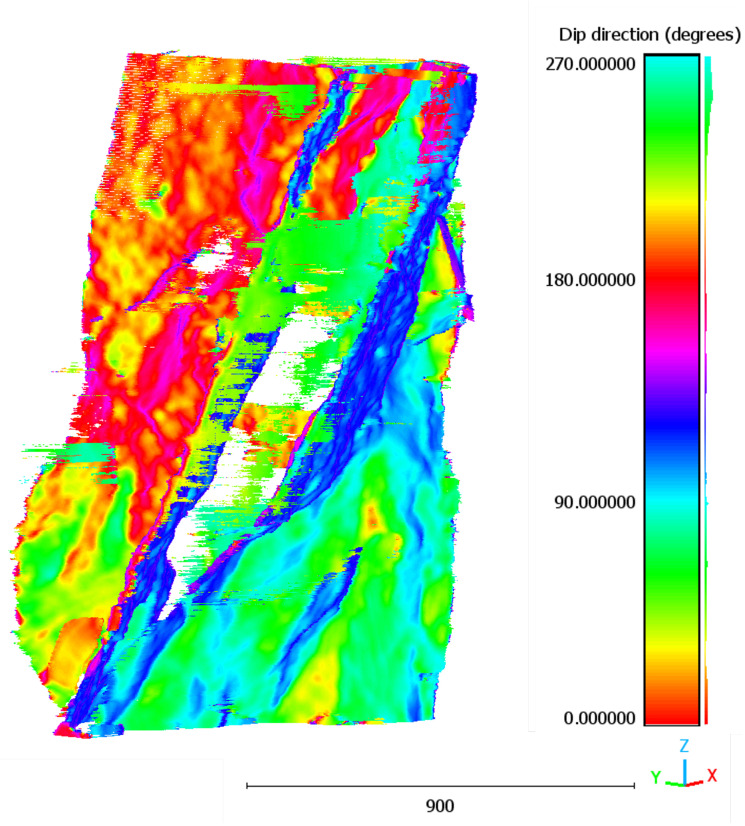
The 3D point cloud of fracture voxels extracted using U-Net method.

Figure 10 shows the point cloud obtained using the deterministic method, which tended to highlight only the most prominent fractures with higher contrast, resulting in a sparser distribution of points. Conversely, Figure 11 illustrates the point cloud derived from the U-Net segmentation masks, revealing a dense and continuous network of fractures, including low-contrast features and subtle branching.

These visual comparisons reinforce the observed differences in fracture coverage, with Method 2 better capturing the continuity and complexity of the network, while Method 1 emphasized precision and low noise, albeit with reduced sensitivity to subtle features.

These findings on detection robustness complement the following geometric and topological evaluation of the extracted fracture networks. Specifically, we computed

- **Fractal Dimension** via 3D box-counting, as an indicator of morphological complexity;
- **Connectivity Index**, defined as the average node degree in the fracture graph, representing structural continuity;
- **Average Cluster Size**, the mean voxel count per connected component, indicating spatial cohesion;
- **Width Estimation Error**, expressed as mean absolute error (MAE) relative to manually validated ground truth data.

The results show that Method 2 (U-Net) had a higher fractal dimension (1.45 vs. 1.32), greater connectivity index (2.8 vs. 2.1), and larger average cluster size (1200 vs. 750 voxels), supporting the notion of improved complexity and continuity in fracture detection. Conversely, Method 1 demonstrated better accuracy in fracture width estimation, with a lower MAE (0.12 mm compared to 0.18 mm), reflecting its sharper localization capability.

This quantitative evidence substantiates the trade-off between precision and completeness observed qualitatively, reinforcing the complementarity of the two approaches.

## 4. Uncertainty Analysis

Uncertainty analysis played a crucial role in quantifying the limitations of the fracture measurements and the reliability of the applied segmentation methods. Several sources of uncertainty were considered, as they could affect the precision of fracture detection and characterization in the X-ray CT data.

### 4.1. Resolution of the Tomography

The spatial resolution of the X-ray tomography system played a key role in the accuracy of fracture detection. The system used in this study had a voxel size of 70.69 μm. This resolution determined the smallest detectable fracture size: any fracture smaller than this voxel size may not have been adequately represented. In particular, fractures that were close to the resolution limit of the system could appear less defined, and their dimensions could be misrepresented. For example, when fractures were located near regions of density transition (such as voids), the system could detect a voxel with an intermediate density, which resulted in a reduced contrast between the fracture and the surrounding material. To quantify this uncertainty, the voxel size needed to be considered as a fraction of the sample’s dimension. When the sample was large, the influence of resolution errors became more significant, especially in the case of fractures located in the central regions of the sample, where X-rays needed to pass through thicker material before reaching the detector.

### 4.2. Phenomena That Degrade Measurement Quality

#### 4.2.1. Contrast and Fracture Recognition

Another significant source of uncertainty was related to the contrast between the fracture and the surrounding material. When the fracture was filled with air or low-density material, the contrast with the surrounding material could be insufficient, making it difficult for both the convolution-based and neural network methods to accurately detect the fracture boundaries. Low contrast in the X-ray data could result in fractures being poorly defined, with blurred edges that hindered accurate segmentation. Moreover, this low contrast could lead to a false identification of material boundaries, especially in regions where the fracture was not well differentiated from the surrounding material. To address this issue, a redundant approach involving scanning from multiple angles was employed to improve fracture detection by capturing more complete structural information. Additionally, convolution-based filtering was applied to reduce the effects of low contrast by smoothing the image and emphasizing local minima corresponding to fracture locations.

#### 4.2.2. Noise and Signal-to-Noise Ratio (SNR)

Noise is an unavoidable factor in tomographic imaging and can arise from multiple sources, such as detector limitations, X-ray scatter, and electronic interference. These types of noise distort fracture boundaries and hinder accurate measurements of their dimensions and positions.

A median filtering technique was applied to the reconstructed volume slices to reduce the impact of high-frequency noise, which could affect segmentation performance. Additionally, a convolution-based filtering method using a rectangular kernel, designed to match the expected geometry of sharp-edged fractures, was implemented. This approach emphasized features consistent with physically plausible fracture morphology and suppressed elements that deviated from such patterns.

By improving the SNR without introducing significant edge blurring, this strategy increased the robustness of fracture detection and segmentation against noise and image degradation. These methods allowed us to consider the main contribution to localization uncertainty as being primarily due to the intrinsic resolution limit of the CT system.

#### 4.2.3. Impact of Filling Materials in Fractures

In rock discontinuities, both open fractures (without filling) and fractures filled with different minerals can occur. In the rocks of the Umbria–Marche Apennines, fracture fillings commonly consist of clays or calcite. These filling materials can reduce the contrast of the fracture and lead to inaccurate segmentation or incorrect fracture characterization. For example, fractures filled with materials of higher density than air may appear as solid structures, even though they are technically voids. This can introduce an additional uncertainty into the fracture measurements, as the system may interpret filled fractures as solid, causing an overestimation of their size. To mitigate this issue, a neural network-based method was employed, which demonstrated improved capability in distinguishing between different material types and compensating for the influence of filling materials [49]. By learning patterns from training data, the neural network was able to detect fractures more accurately, even in the presence of such materials. Each of these factors contributed to the overall uncertainty, and the applied methods—including convolution filtering, redundancy from multiple scanning angles, and neural network-based segmentation—were intended to minimize their impact and enhance the reliability of the measurements.

#### 4.2.4. Artifacts from Reconstruction (Radon Transform)

The tomographic reconstruction process, specifically the Radon transform, could introduce reconstruction artifacts that affected the quality of the image and the accuracy of the fracture measurements. These artifacts were particularly noticeable in the central regions of the sample, where the X-rays needed to pass through a larger thickness of material before reaching the detector. The resulting intensity distortions were primarily attributed to beam hardening effects and, to a lesser extent, X-ray scatter. Due to the limited angular sampling inherent in the Radon transform, regions exhibiting sharp density contrasts, such as low-density fractures or voids embedded in a higher-density matrix, could suffer from incomplete reconstruction, affecting the accuracy of fracture delineation, particularly in the central parts of the sample.

### 4.3. Role of Convolution and Compensation Strategies

Several sources of uncertainty discussed above, such as resolution limits, low contrast, and noise, were effectively mitigated through the application of convolution filtering. In particular, the use of a rectangular convolution kernel, based on a geometric model of the fracture as a sharp-edged discontinuity, enhanced features that were physically consistent with expected fracture shapes, while suppressing geometries that did not correspond to real morphological structures.

Unlike sub-pixel interpolation algorithms, the rectangular convolution did not increase resolution per se. Instead, it improved the robustness of the detection technique against noise, reconstruction artifacts, and other forms of degradation. This robustness was particularly relevant when the signal was weak or when fractures were located in noisy or low-contrast regions.

The selection of the rectangular convolution kernel was guided by the actual geometry of the fractures in the sample. Although the cross-section of a fracture in a single tomographic slice could resemble a Gaussian profile—mainly due to smoothing effects inherent in the reconstruction process—such a representation, while producing higher numerical correlation, did not accurately reflect the true fracture shape. A Gaussian kernel, although potentially useful for sub-pixel refinement, was less effective in isolating real fractures from other structural features, potentially increasing the number of false positives.

To compensate for shading effects observed in the central regions of the sample—resulting from increased X-ray path length and reconstruction limitations—a normalization step was applied by subtracting the mean intensity value from each tomographic slice. This correction improved the homogeneity of the density distribution and enhanced fracture visibility throughout the volume.

In order to avoid introducing additional uncertainty, sample displacement during scanning was not employed. Although such techniques may improve angular coverage, they risk reducing effective resolution and introducing motion-related artifacts.

#### Quantitative Uncertainty Estimation

To quantitatively evaluate the uncertainty associated with the fracture detection methods, we conducted multiple segmentation runs while varying critical parameters such as threshold values and filtering criteria. In particular, the segmentation threshold was varied between 0.2 and 0.5 (normalized intensity units) in increments of 0.05, and the corresponding number of detected fracture voxels was recorded.

For the deterministic method, the number of fracture voxels ranged from 15,000 at a threshold of 0.2 to 9800 at 0.5, resulting in a relative variation of approximately 35%. However, the quality of the results was found to be strongly dependent on selecting the correct threshold value. A small deviation from the optimal threshold often led to significant misclassifications, especially in noisy or low-contrast regions. This high sensitivity to thresholding highlighted a limitation of the deterministic approach, despite its overall robustness under fixed parameters.

In contrast, the U-Net method, although also threshold-based, exhibited more stable behavior. The number of detected fracture voxels varied from 8200 to 6500 over the same threshold range, corresponding to a variation of about 21%. While this appeared to reflect greater robustness in voxel count, in practice, the segmentation quality remained consistently high over a broader range of thresholds. This indicated that the U-Net method was less critically dependent on precise threshold selection. Although it tended to over-segment and include some false positives, this was effectively mitigated at a later stage by applying convolution filtering to refine the fracture geometry.

The variability in fracture voxel counts, summarized by the coefficient of variation (CV), was 14.5% for the deterministic method and 7.8% for the U-Net method, supporting the notion of greater parameter robustness in the latter, but also reinforcing the operational flexibility of the former.

To further assess sensitivity to noise, controlled Gaussian noise was artificially introduced into the tomographic datasets. The deterministic method exhibited an average width estimation variation of 0.04 mm, while the U-Net method showed a larger variation of 0.11 mm, confirming its increased susceptibility to noise and imaging artifacts.

False positive and false negative rates were also computed with respect to manually annotated ground truth data. The deterministic method yielded a lower false positive rate (5.3%) but a higher false negative rate (17.6%), consistent with its conservative detection strategy. In contrast, the U-Net method produced a higher false positive rate (13.8%) and a lower false negative rate (9.2%), reflecting its tendency to detect a broader range of fracture-like structures, including more ambiguous voxels.

**Figure 12:**
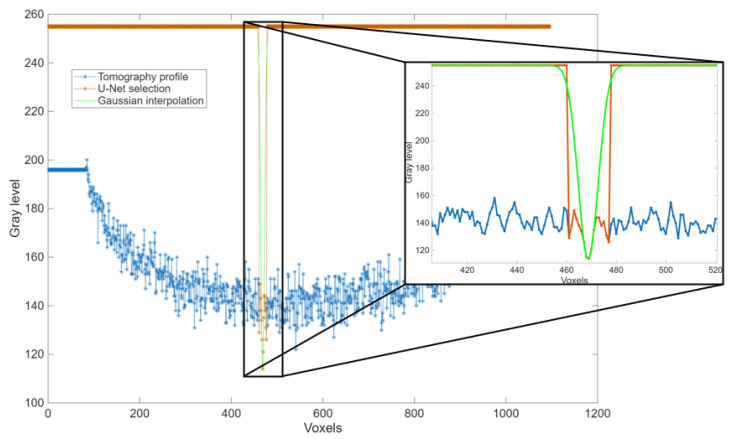
Example of a cross-section segmented using the U-Net method. The network successfully captured the fracture structure (in red) but tended to slightly over-segment in low-contrast regions. These regions were later corrected during convolutional filtering (in green).

This behavior is illustrated in Figure 12, where the U-Net segmentation (in red) captures the fracture accurately but also includes surrounding low-contrast regions that are not part of the true fracture. These over-segmented areas were subsequently corrected during the convolutional post-processing stage (result shown in green), which retained only the most geometrically coherent and relevant features. As a result, the initial over-segmentation was effectively mitigated, and the impact of false positives on the final analysis remained minimal.

In addition to voxel-based variability, we also considered sub-voxel localization accuracy. Although the spatial resolution of the tomographic volume was constrained by the voxel size (70.69 μm), the deterministic method enabled more precise fracture localization by analyzing grayscale intensity profiles along orthogonal directions. This allowed for identifying fracture-related intensity minima with a spatial accuracy of at least 1/20 of a voxel, i.e., around 3.5 μm. While this did not increase the native resolution of the imaging system, it enhanced the localization capability, which was not achievable with the binary masks produced by the U-Net method, the spatial accuracy of which was inherently limited by the voxel grid.

These combined strategies for fracture extraction contributed significantly to minimizing slice degradation and reconstruction artifacts. Consequently, the primary source of uncertainty in fracture localization could be attributed to the resolution of the tomographic system. Based on a voxel size of 70.69 μm, the localization uncertainty was conservatively estimated to be ±35.35 μm.

## 5. Discussion

The application of two distinct methodologies for fracture detection—one based on deterministic image processing and the other on deep learning—highlighted complementary strengths and limitations that are particularly relevant in geological and geomechanical applications.

The deterministic method proves highly effective in identifying high-contrast, open fractures with minimal background interference. Its reliance on physical signal interpretation and peak detection provides localized precision, especially useful for quantifying sharp discontinuities. However, its performance declines in cases where fractures are partially filled or where the density contrast is insufficient to create sharp local minima in the intensity profiles.

Conversely, the U-Net-based approach excels in capturing spatially continuous and complex fracture geometries. Its capacity to learn from annotated data allows it to generalize even to low-contrast features and filled discontinuities. Nevertheless, it suffers from a higher sensitivity to noise and false positives, particularly when the segmentation threshold is sub-optimally chosen. Moreover, the success of the method depends on the quality and diversity of the training data, which may limit its generalizability to different lithologies or acquisition conditions. The use of U-Net for this case study was satisfactory for the detection of cracks in the material despite the fact that the segmentation output overestimated the size of the defect. However, this overestimation allowed for the identification of as many regions crossed by the crack as possible, particularly small or partial defects that would normally be difficult to detect using deterministic approaches.

An interesting outcome of this study is the possibility of integrating the two methods in a hybrid framework. For instance, U-Net segmentation could serve as a first-pass detector of potential fracture zones, while deterministic peak detection could refine the fracture localization within those zones. Such an approach could combine the robustness and coverage of deep learning with the precision of deterministic signal analysis.

The uncertainty analysis further supports this dual approach. Different methods show different sensitivities to noise, resolution limits, and reconstruction artifacts. By merging their outputs, it may be possible to attenuate these individual weaknesses and improve overall fracture detection reliability.

Our findings are consistent with previous studies that highlight the potential of deep learning for enhancing fracture detection in tomographic data. For instance, the improved continuity and coherence of the fracture segments obtained through the U-Net approach align with the results reported by Lu et al. [46] and Byun et al. [48], who also emphasized the advantages of convolutional networks in identifying complex discontinuities under variable contrast conditions. Furthermore, the observed limitations in detecting filled fractures using deterministic methods are in line with what was reported by Afshari et al. [50], where low contrast between matrix and vein material posed significant challenges for classical image analysis. These comparisons confirm the broader applicability of hybrid or machine learning-based approaches in geotechnical and structural geology contexts.

Future work should aim to test the robustness of the combined methodology on datasets acquired under different scanning conditions or involving samples with different lithological properties. Furthermore, more advanced post-processing techniques, such as graph-based analysis of point clouds or fracture network topology, could provide deeper geological insights beyond mere detection.

In terms of practical applications, our methodology is well-suited for geological and geotechnical tasks such as fault characterization, reservoir evaluation, and stability analysis in rock mechanics, as reported by many authors [50,51,52]. Matlab algorithms are computationally efficient, easily reproducible, and inexpensive, making them accessible for rapid analyses by other researchers, accelerating the analysis and characterization of fracture networks that can be directly used for modeling purposes [50]. However, it remains essential to validate the results with field data or classical measurements performed on the samples, as emphasized by Mammoliti et al. [11].

One of the main limitations requiring further research is the low contrast between certain discontinuity types, such as calcite-filled veins embedded in a carbonate matrix, which appear very similar and are difficult to distinguish using tomography.

## Data Availability

Research data are available upon request.
